# Probing Arginine Side-Chains and Their Dynamics with Carbon-Detected NMR Spectroscopy: Application to the 42 kDa Human Histone Deacetylase 8 at High pH**

**DOI:** 10.1002/anie.201209385

**Published:** 2013-02-10

**Authors:** Nicolas D Werbeck, John Kirkpatrick, D Flemming Hansen

**Affiliations:** Institute of Structural and Molecular Biology, University College LondonGower Street, WC1E 6BT, London (UK)

**Keywords:** arginine, histone deacetylases, NMR spectroscopy, protein dynamics, spin relaxation

Arginine has a unique role among the 20 standard proteinogenic amino acids. In contrast to other charged amino acid side-chains the charge of arginine side-chains is relatively unaffected by the surrounding environment.[1] As a consequence, arginine provides nature with a reliable means of placing a positive charge at any required position in a biomolecule, such as in active sites or at protein–ligand interfaces. Few methods are currently available to probe charged side-chains and their dynamics under physiological conditions, although such methods are pivotal in order to elucidate protein–ligand interactions and important to relate molecular motions to biomolecular functions.

NMR spectroscopy is a powerful technique to probe protein environments and characterize dynamics of proteins at atomic resolution.[2] For amino acids with large side-chains, such as arginine and isoleucine, the terminal moieties are nearly uncoupled from the backbone.[3] Methods that probe large methyl-bearing side-chains have therefore been successfully applied to characterize the function of enzymes and macromolecular machines.[4] Techniques that probe charged side-chains are currently limited,[5] despite their importance for enzyme catalysis and protein–ligand interactions. With regard to probing arginine and lysine side-chains, conventional methods are often impeded by rapid exchange of the detected protons with the bulk solvent, which can lead to extensive line-broadening and effectively undetectable signals at and above physiological pH. To alleviate this problem, Mulder and co-workers[6] introduced an elegant approach relying on detection of aliphatic protons. Whilst this method circumvents the rapid exchange and allows measurements of the ^15^N_ε_ chemical shift of arginine, it is less beneficial for nuclear spin relaxation measurements and for studying larger proteins, where the two bound protons enhance the transverse relaxation of the aliphatic carbons.

Herein we describe an NMR pulse scheme for probing arginine side-chains and their dynamics at neutral-to-high pH by carbon-detected ^13^C_ζ_–^15^N_ε_ correlated spectra (Figure 1). Magnetization is transfered from ^13^C_ζ_ to ^15^N_ε_ via the scalar coupling, while transfers from ^13^C_ζ_ to ^15^N_η_ are avoided by applying a selective ^15^N_ε_ inversion pulse in the INEPT transfer steps. After the initial INEPT transfer, the magnetization is proportional to 2*C*_ζ,z_*N*_ε,z_, which allows spin relaxation measurements and quantification of the squared order parameter, *S*^2^, that reports on the motions of the arginine side-chain.[7] Here, *S*^2^≈0 indicates that the motion of the side-chain is completely uncoupled from the overall molecular tumbling, whereas *S*^2^≈1 shows that the side-chain is rigid with respect to the overall molecular frame.

**Figure 1:**
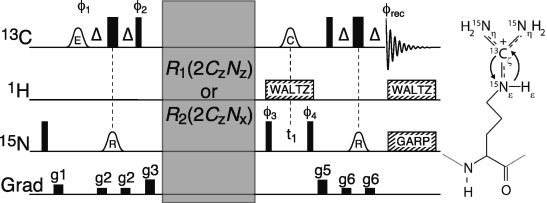
The ^13^C_ζ_–^15^N_ε_ HSQC pulse scheme for probing arginine side-chains at neutral-to-high pH. The carrier positions are ^13^C: 159 ppm, ^15^N: 84 ppm (78 ppm decoupling), and ^1^H: 7 ppm. Narrow bars represent 90° pulses and wide bars represent 180° pulses. The delay Δ is 1/(4*J*_Cζ–Nε_)=12.5 ms. Shaped pulses are represented by bell-shapes with letters specifying the shape (R: RE-BURP, E: E-BURP-2,[8] C: smoothed CHIRP[9]). Phases are *x* unless stated otherwise. The phase cycle used is ϕ_1_: 4(*x*), 4(−*x*), ϕ_2_: *y*, ϕ_3_: *x*,−*x*, ϕ_4_: 2(*x*), 2(−*x*), ϕ_rec_: *x*, 2(−*x*), *x*, −*x*, 2(*x*), −*x*. Decoupling sequences are represented by striped boxes indicating the type of decoupling: WALTZ64[10] (4 kHz), GARP4[11] (0.7 kHz). Gradients of 1 ms are represented by black rectangles and applied with strength of g1: 9.5 G cm^−1^, g2: 3.9 G cm^−1^, g3: 26.2 G cm^−1^, g5: 18.4 G cm^−1^, g6: 7.2 G cm^−1^. Modules embedded in the gray box for measuring the anti-phase relaxation rates *R*_2_(2*C*_z_*N*_x_) and *R*_1_(2*C*_z_*N*_z_) are described in the the Supporting Information.

As a first application we probed the arginine side-chains of T4 lysozyme L99A (T4L L99A; 18 kDa) shown in Figure 2 a. T4L L99A is stable at pH 5.5[12] and therefore provides an opportunity for a comparison of the carbon-detected methodology proposed here with the conventional proton-detected methods. The ^13^C_ζ_–^15^N_ε_ spectrum of T4L L99A, Figure 2 b, shows many well-dispersed peaks and a chemical shift dispersion similar to the corresponding ^1^H_ε_–^15^N_ε_ spectrum (Supporting Information). As expected, the signal/noise ratios of the disperse peaks of the ^13^C_ζ_–^15^N_ε_ spectrum are approximately a factor of 30 less than the corresponding peaks of the ^1^H_ε_–^15^N_ε_ spectrum obtained with the same recording time. The proton-detected experiments therefore remain preferable at pH below ca. 6, whereas at higher pH the disadvantage of the longer recording time of the ^13^C_ζ_–^15^N_ε_ spectrum is often outweighed by the fact that the ^1^H_ε_–^15^N_ε_ spectrum provides very limited information.

**Figure 2:**
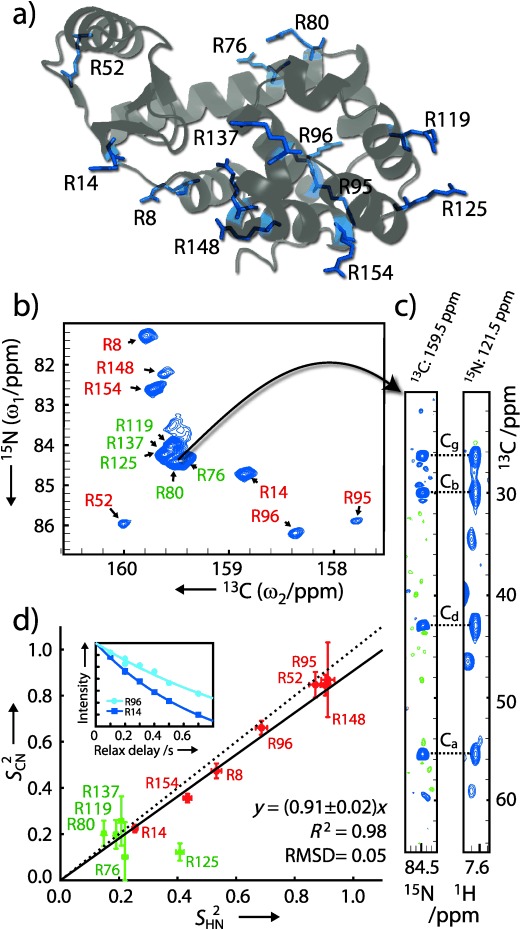
a) Structure of T4L L99A (PDB: 1L90[13]). b) ^13^C_ζ_–^15^N_ε_ spectrum of T4L L99A obtained at 16.4 T (176 MHz ^13^C frequency) and 298 K with the pulse scheme shown in Figure 1. c) A chemical shift assignment is exemplified with the ^13^C_ζ_–^15^N_ε_ resonance of R80 assigned from the carbon-detected 3D CCNeCz-TOCSY (left; Supporting Information), the 3D CC(CO)NH-TOCSY[14] (right), and the backbone assignment[12] of T4L L99A. d) Comparison of order parameters derived from the carbon-detected experiments, *S*^2^_CN_, with those derived from proton-detected ^1^H_ε_–^15^N_ε_ experiments, *S*^2^_HN_. The order parameters, *S*^2^_CN_, were derived from the *R*_1_(2*C*_z_*N*_z_), *R*_2_(2*C*_z_*N*_x_), and *R*_1_(*C*_z_) relaxation rates as described in the Supporting Information. The inset shows examples of decay curves used to determine the relaxation rates: R96: *R*_1_(2*C*_z_*N*_z_)=0.78±0.06 s^−1^, R14: *R*_1_(2*C*_z_*N*_z_)= 1.32±0.02 s^−1^. The wide range of order parameters observed for the arginine side-chains of T4L L99A suggests possible motions around the four side-chain dihedral angles, in agreement with results obtained for methyl-bearing side-chains.[15] RMSD=root mean-square deviation.

Spin relaxation rates were obtained from a series of ^13^C_ζ_–^15^N_ε_ spectra and order parameters were subsequently calculated for the arginine side-chains of T4L L99A. Figure 2 d shows a good agreement when these order parameters are compared to the corresponding order parameters, *S*^2^_HN_, obtained using proton-detected experiments.[16] For the seven isolated peaks (R8, R14, R52, R95, R96, R148, R154), we obtain RMSD(*S*^2^_CN_, *S*^2^_NH_)=0.05. Including the peaks of the more crowded region (green in Figure 2 d) gives the same general picture, despite a higher uncertainty of these parameters.

The human histone deacetylase 8 (HDAC8) catalyzes the de-acetylation of lysine side-chains in cells and helps to balance the acetylation state of proteins.[17] HDAC8 is a 42 kDa metalloenzyme with 11 arginines, at least one of which is crucial for activity.[18] Activity assays and purifications have been established at pH≈8 and the unliganded form of HDAC8 appears to be unstable at pH lower than ca. 7, based on 2D NMR. Thus, the ^1^H_ε_–^15^N_ε_ spectrum is not applicable to probe the arginine side-chains of HDAC8, as shown in Figure 3 a, and these side-chains must be probed independently of the ^1^H_ε_ spin. Figure 3 a and 3 b show a ^1^H_ε_–^15^N_ε_ and a ^13^C_ζ_–^15^N_ε_ spectrum, respectively, of HDAC8 at pH 8.2 and clearly demonstrate that under these conditions the ^13^C_ζ_–^15^N_ε_ spectrum reveals many more features than the corresponding ^1^H_ε_–^15^N_ε_ spectrum.

**Figure 3:**
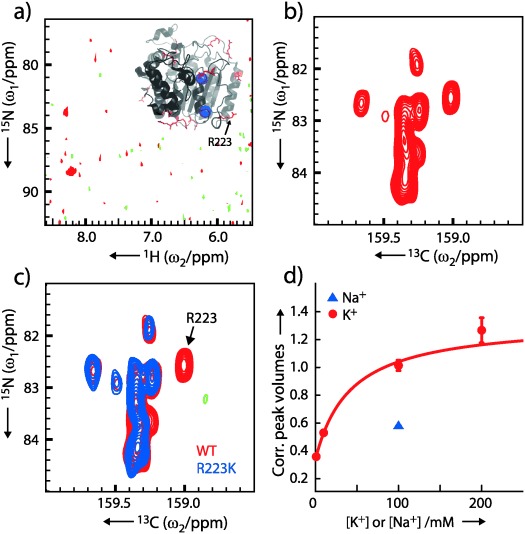
a) HDAC8 ^1^H_ε_–^15^N_ε_ TROSY spectrum recorded for 11 h at 298 K and 16.4 T (0.3 mm protein, pH 8.2, 10% ^2^H_2_O) and structure of HDAC8 (PDB 2V5W[20]). A maximum of two of the 11 arginines can be identified in the ^1^H_ε_–^15^N_ε_ spectrum due to rapid exchange of ^1^H_ε_ with the bulk solvent. b) Wild-type HDAC8 ^13^C_ζ_–^15^N_ε_ spectrum recorded for 15 h, ≈99.9 % ^2^H_2_O c) Overlay of ^13^C_ζ_–^15^N_ε_ spectra of wild-type HDAC8 (red) and R223K HDAC8 (blue). R223 can be assigned easily since this peak is absent in the spectrum of the R223K mutant. d) The corrected R223 peak volumes in the ^13^C_ζ_–^15^N_ε_ spectra as a function of the concentration of K^+^ (red circles) and Na^+^ (blue triangles). The line represents a fit of a hyperbolic binding curve to the data with *K*_D_=42±9 mm. This model assumes that R223 probes only one of the K^+^ binding sites and that the volume of the R223 peak is proportional to the population of the K^+^-bound state.

Potassium binding has recently been shown to regulate the activity of HDAC8.[19] Crystal structures of HDAC8[20] show that the C_ζ_ of R223 is ca. 12 Å from one of the two potassium binding sites, which allows us to investigate the wider consequences on the side-chain packing of K^+^ binding. The peak of R223 was assigned by mutation of R223 to lysine, which caused the disappearance of an isolated peak as seen in Figure 3 c. Other minor perturbations are observed in the spectrum as expected for this allosteric enzyme and also observed for the assignment of other side-chain chemical shifts.[4] At low concentrations of K^+^ the R223 peak is hardly visible, whereas its intensity increases as the concentration of K^+^ is increased, Figure 3 d. On the contrary, addition of Na^+^ does not lead to the same increase in intensity, thus attesting that the effect observed is due to specific K^+^ binding rather than electrostatic stabilization of the protein. No extra isolated peak appears at low concentrations of K^+^, suggesting that the R223 side-chain of the potassium-free form is either disordered or undergoing chemical exchange, such that the corresponding peak is located in the random-coil region or broadened beyond detection, respectively. Although the four titration points obtained here are not sufficient to determine an accurate dissociation constant for K^+^, an initial estimation of *K*_D_≈40 mm is in agreement with a previously determined *K*_D_ from activity assays.[19] Overall, our arginine data show that binding of K^+^, which activates HDAC8, affects not only the backbone binding site, but also changes the side-chain packing beyond the binding site.

In general, the side-chains of amino acids probe a different environment from that of the backbone, and charged side-chains probe a different environment from that of hydrophobic side-chains. It has now become clear that probing methyl-bearing side-chains provides very important information about protein function and dynamics.[21] The methodology presented here extends the utility of side-chains as probes of structure and dynamics to include the charged arginine side-chain, and in the context of proteins at physiological pH provides an avenue for characterizing arginine side-chain interactions at a level of detail that has largely been, until now, reserved for applications to methyl-bearing side-chains.

## Experimental Section

Sample preparation: U-[^13^C,^15^N,^2^H] T4L L99A, wild-type HDAC8, and R223 K HDAC8 were expressed and purified as described in the Supporting Information. All carbon-detected NMR experiments were performed on a Bruker Avance III 700 MHz (16.4 T) spectrometer using an HCN inverse cryoprobe (CP-TCI). A comprehensive list of all experiments including sample details, experimental conditions, and recording parameters is given in the Supporting Information. NMR spectra were processed with NMRpipe[22] and relaxation rates were obtained using FuDA.[23] Order parameters were obtained using model-free approaches[7] as described in the Supporting Information. For the potassium titration of HDAC8 peak volumes were processed as described in the Supporting Information.

## Notes

Supporting information for this article (including specific experimental procedures, sample preparation, and NMR measurements) is available on the WWW under http://dx.doi.org/10.1002/anie.201209385.
