# Supplementary material for: Probing Arginine Side-Chains and Their Dynamics with Carbon-Detected NMR Spectroscopy: Application to the 42 kDa Human Histone Deacetylase 8 at High pH
Source: Angew Chem Int Ed Engl. 2013 Feb 10;52(11):3145–7. doi: 10.1002/anie.201209385 (PMC4016738; doi:10.1002/anie.201209385)
Supplement: Supplementary file 1 [file anie0052-3145-sd1.pdf]

Angewandte Chemie
Eine Zeitschrift der Gesellschaft Deutscher Chemiker

Supporting Information

© Wiley-VCH 2013

69451 Weinheim, Germany

Probing Arginine Side-Chains and Their Dynamics with Carbon-Detected NMR Spectroscopy: Application to the 42 kDa Human Histone Deacetylase 8 at High pH**

*Nicolas D. Werbeck, John Kirkpatrick, and D. Flemming Hansen**

anie_201209385_sm_miscellaneous_information.pdf

Theoretical aspects of probing arginine side-chain dynamics

Determining arginine $^{15}N_\varepsilon$ spin relaxation rates

The nuclear spin-relaxation of $^{15}N_\varepsilon$ can be treated as described previously[1] and is similar to the relaxation of the amide nitrogen in the protein backbone. The relaxation of $^{15}N_\varepsilon$ is caused by the $^{15}N_\varepsilon$-$^{1}H_\varepsilon$ dipole-dipole interaction and the chemical shift anisotropy (CSA), $\Delta\sigma_{N\varepsilon}$ = –114 ppm[2], assuming axial symmetry of the CSA tensor. Due to the weak scalar coupling between $^{13}C_\zeta$ and $^{15}N_\varepsilon$ ($^{1}J_{C\zeta\text{-}N\varepsilon} \approx 20$ Hz), we prefer to derive the motional parameters from the anti-phase relaxation rate constants, $R_1(2C_zN_z)$ and $R_2(2C_zN_x)$. By adopting this approach instead of measuring the in-phase nitrogen relaxation rates, we can limit the time the magnetization spends in the transverse plane, because the extra refocusing step that would be required can be omitted and additional loss of coherence is avoided. As shown below, the measurement of anti-phase relaxation rates does not affect the accuracy of the derived motional parameters. The contributions from cross-correlations involving $^{15}N_\varepsilon$ and $^{13}C_\zeta$ can be neglected because of the weak $^{15}N_\varepsilon$-$^{13}C_\zeta$ dipole-dipole interaction (see below) and we therefore obtain:

$$R_2(N_x) \approx R_2(2C_zN_x) - R_1(C_z) \quad \text{(S1)}$$

$$R_1(N_z) \approx R_1(2C_zN_z) - R_1(C_z) \quad \text{(S2)}$$

where $R_2(N_x)$ and $R_1(N_z)$ are the transverse and longitudinal $^{15}N_\varepsilon$ relaxation rates defined below and $R_1(C_z)$ is the longitudinal relaxation rate of $^{13}C_\zeta$, which we determine experimentally. Apart from possible interference from cross-correlation effects, the above approximations neglect higher-frequency spectral density terms in the dipolar contributions to the relaxation rates. These approximations are reasonable due to the weak $^{15}N_\varepsilon$-$^{13}C_\zeta$ dipole-dipole interaction, as shown below.

Theoretical simulations were used to verify the approximations made in Eqs S1 and S2 and in particular to investigate the effect of the cross-correlation between the $^{15}N_\varepsilon$-$^{13}C_\zeta$ dipole-dipole and $^{13}C_\zeta$ CSA or $^{15}N_\varepsilon$ CSA relaxation mechanisms. Since $R_2(N_x)$ in most instances is significantly larger than $R_1(N_z)$ a verification of the approximation made in Eq. S2 also verifies Eq. S1. To investigate the cross-correlation between the $^{15}N_\varepsilon$-$^{13}C_\zeta$ dipole-dipole and $^{13}C_\zeta$ CSA or $^{15}N_\varepsilon$ CSA relaxation mechanisms we considered a basis of four normalized operators in the product operator formalism: $\{E/2, C_z, N_z, 2C_zN_z\}$, where E is the unity operator, C_z is the longitudinal operator for $^{13}C_\zeta$, N_z is the longitudinal operator for $^{15}N_\varepsilon$, and $2C_zN_z$ is the longitudinal two-spin order operator. Auto relaxation rates and transition rates between the four operators were calculated using a $^{13}C_\zeta$-$^{15}N_\varepsilon$ distance of 1.33 Å, a $^{13}C_\zeta$ axial CSA of 78 ppm[3], and a $^{15}N_\varepsilon$ CSA of –114 ppm.[2] A contribution of 0.3 s^{-1} was added to the auto-relaxation rates of C_z and $2C_zN_z$ to account for the $^{13}C_\zeta$–$^{1}H_{\varepsilon,\eta}$ dipolar interactions, and the

relaxation of $^{15}N_\varepsilon$ caused by the $^{1}H_\varepsilon$ was explicitly added to the auto-relaxation rates of N_z and $2C_zN_z$. The experimentally extracted relaxation rates $R_1(C_z)$ and $R_1(2C_zN_z)$ were simulated by (1) integrating the homogeneous master equation[4] over the corresponding relaxation delays[5], T_{relax} in Figures S2 & S3, for values of T_{relax} used experimentally and (2) fitting the resulting intensities to single exponential decays. As a measure of the accuracy of Eq. S2 we have plotted in Figure S1 the fractional error introduced by our assumptions: $[R_1(N_z) - \{R_1(2C_zN_z) - R_1(C_z)\}]/R_1(N_z)$, where $R_1(N_z)$ is the auto-relaxation rate of N_z calculated above. Overall, the total error introduced by neglecting the higher-frequency spectral density terms in the dipolar contributions to the auto-relaxation and cross-correlation between the $^{15}N_\varepsilon$-$^{13}C_\zeta$ dipole-dipole and $^{13}C_\zeta$ CSA or $^{15}N_\varepsilon$ CSA relaxation mechanisms is always less than 4.5 %.

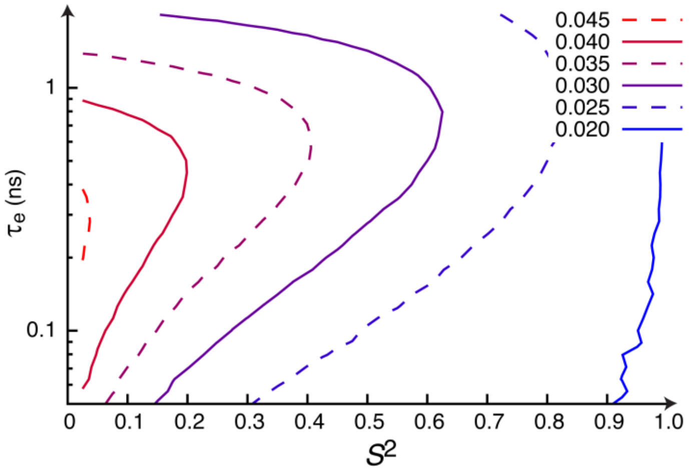

Figure S1: Contour plot of fractional error introduced by the assumption made in Eq. S2 for probing arginine side-chain dynamics. The fractional error, $[R_1(N_z) - \{R_1(2C_zN_z) - R_1(C_z)\}]/R_1(N_z)$ is shown for different values of the order parameter, S^2, and local correlation times, τ_e, assuming an overall rotational correlation time of 11.6 ns and a static magnetic field strength of 16.4 T.

Extraction of motional parameters

In general, motions of a specific bond-vector on the pico-to-nanosecond timescale are accessible by experimentally measuring nuclear relaxation rates that, in turn, can be interpreted in terms of meaningful physical parameters using different model-dependent[6] and model-independent treatments.[7] For a given IS spin-system, such as an ^{1}H-^{15}N spin-pair, the longitudinal relaxation rate, $R_1(N_z)$, the transverse relaxation rate, $R_2(N_x)$, and the steady-state nuclear Overhauser effect, {^{1}H}-^{15}N NOE, can be expressed in terms of the spectral density function, $J(\omega)$[8]:

$$R_1(N_z) = \frac{1}{4}d^2\big(3J(\omega_N) + 7J(\omega_h)\big) + c^2 J(\omega_N) \quad \text{(S3)}$$

$$R_2(N_x) = \frac{1}{8}d^2(4J(0) + 3J(\omega_N) + 13J(\omega_h)) + \frac{1}{6}c^2(4J(0) + 3J(\omega_N)) + R_{ex} \quad \text{(S4)}$$

$$\{\,^{1}\mathrm{H}\} - {}^{15}\mathrm{N\ NOE} = 1 + \frac{1}{4R_1(N_z)}d^2(\gamma_H/\gamma_N)(5J(\omega_h)), \quad \text{(S5)}$$

where

$$c = \omega_N\, \Delta\sigma_N/\sqrt{3} \quad \text{(S6)}$$

$$d = (\mu_0 h \gamma_N\, \gamma_H / 8\,\pi^2)\langle 1/r_{NH}^3\rangle, \quad \text{(S7)}$$

and $J(0)$, $J(\omega_N)$ and $J(\omega_h)$ are the values of the spectral density function evaluated at a frequencies of 0 rad s^{-1}, ω_N, and ω_h, respectively, where ω_N is the ^{15}N Larmor frequency and $\omega_h = 0.87\omega_H$ is the effective proton Larmor frequency.[9] The contribution to the transverse relaxation that originates from fluctuations of the Zeeman Hamiltonian due to chemical exchange processes is R_{ex}. In Eqs. S6 and S7, $\Delta\sigma_N$ is the chemical shift anisotropy (in ppm), assuming axial symmetry for the ^{15}N chemical shift tensor ($\Delta\sigma_N$ = –160 to –172 ppm[1] for backbone ^{15}N and $\Delta\sigma_{N\varepsilon}$ = –114 ppm for arginine side-chain $^{15}N_{\varepsilon}$[2]), μ_0 is the permeability of free space, h is Planck's constant, γ_H and γ_N are the magnetogyric ratios of ^{1}H and ^{15}N, respectively, and r_{NH} = 1.02–1.04 Å is the N-H bond length.[1]

The spectral density function, $J(\omega)$, can be expressed in the model-free formalism as a function of the generalized order parameter, S^2, the overall rotational correlation time of the protein, τ_R, and a time-constant for the local motion, τ_e[7]:

$$J(\omega) = \frac{2}{5}\left[\frac{S^2\tau_R}{1+\omega^2\tau_R^2} + \frac{(1-S^2)\tau_{e'}}{1+\omega^2\tau_{e'}^2}\right], \quad \text{(S8)}$$

where $\tau_{e'}^{-1} = \tau_R^{-1} + \tau_e^{-1}$. The overall rotational correlation time, τ_R, is determined below from the backbone $R_1(N_z)$ and $R_2(N_x)$ rates using only those residues with limited internal dynamics and no contributions to the transverse relaxation from chemical exchange.

NMR Pulse sequences

Spin Relaxation Measurements

All the carbon-detected pulse sequences for probing arginine side-chains presented here are based on the template $^{13}C_\zeta$-$^{15}N_\varepsilon$ sequence shown in Figure 1. Specifically, transfers from $^{13}C_\zeta$ to $^{15}N_\eta$ are avoided by applying a selective RE-BURP[10] centred at 84 ppm, and with a length of 5.1 ms at 16.4 T. Composite-pulse proton (deuterium) decoupling is applied during the indirect chemical shift evolution to remove the $^{15}N_\varepsilon$-$^{1}H_\varepsilon$ ($^{15}N_\varepsilon$-$^{2}H_\varepsilon$) coupling evolution in samples dissolved in H_2O (D_2O) as suggested previously.[11] Proton decoupling is applied during acquisition to remove the small $^{13}C_\zeta$-$^{1}H_{\varepsilon,\eta}$ scalar coupling and ^{15}N decoupling is applied to remove $^{13}C_\zeta$-$^{15}N_{\varepsilon,\eta}$ coupling evolution. Carbon decoupling during the indirect chemical shift evolution of $^{15}N_\varepsilon$ is implemented using a broadband adiabatic sweep with a sweepwidth of 80 kHz and centred at 100 ppm[12], which decouples both the $^{15}N_\varepsilon$–$^{13}C_\zeta$ and the $^{15}N_\varepsilon$-$^{13}C_\delta$ couplings. The modules included in Figure 1 to allow for relaxation measurements are shown in Figure S2.

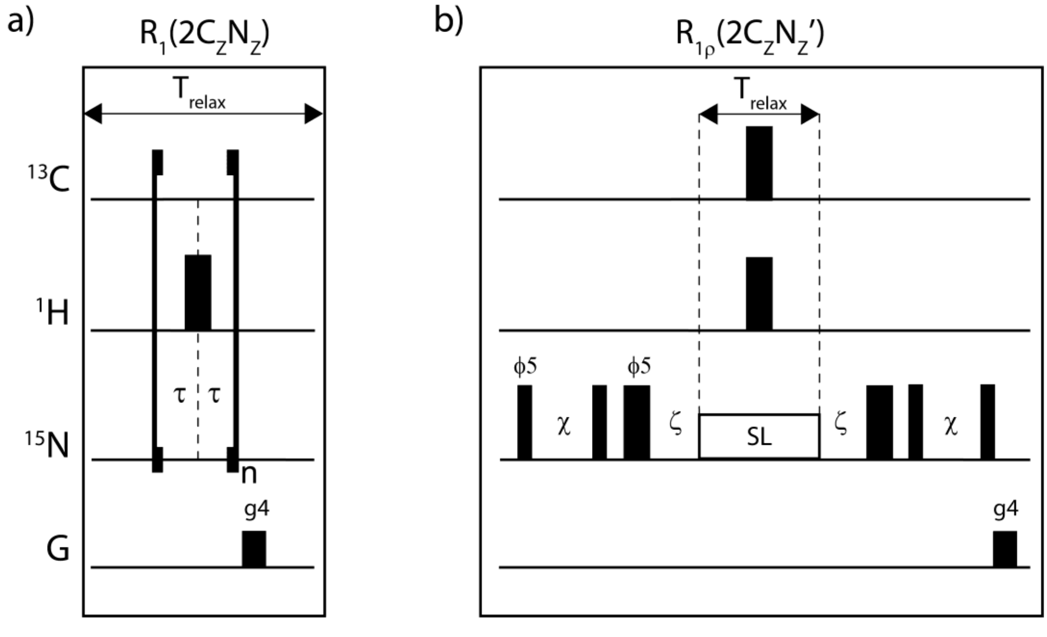

Figure S2: **(a)** Module embedded in Figure 1 for measuring N_ε-relaxation $R_1(2C_zN_z)$, T_{relax} indicates a variable relaxation delay time: $T_{relax} = 2N\tau$, where N is an integer and τ = 50 ms. The phase cycle is: ϕ_1:x, ϕ_2: 4(y),

4(−y), ϕ_3: x, −x ϕ_4: 2(x), 2(−x), ϕ_{rec}: x, 2(−x), x, −x, 2(x), −x. The gradient g4 is a smoothed-square gradient of 1 ms with a strength of 22.8 G/cm. **(b)** Nitrogen anti-phase relaxation $R_{1\rho}(2C_zN_z')$ in the rotating frame used to calculate $R_2(N_x)$ is measured by applying a spin-lock (SL) during T_{relax}. The spin-lock field strength is ν_{SL} = 2.0 kHz. The flanking alignment sequences[13] have the following delays $\chi = 1/(2\pi\nu_{SL}) - 4\tau_{90}^{N}/\pi$ and $\zeta = 2\tau_{90}^{N}/\pi$, where τ_{90}^{N} is the ^{15}N 90° pulse width. The phase cycles are as follows: ϕ_1:x , ϕ_2: y, ϕ_3: x, −x ϕ_4: 2(x), 2(−x), ϕ_5: 4(y), 4(−y), ϕ_{rec}: x, 2(−x), x, −x, 2(x), −x.

Figure S2a shows the element implemented in the pulse sequence for measuring the relaxation rate $R_1(2C_zN_z)$ of the longitudinal two-spin order coherence $2C_zN_z$. Cross-correlation effects between the $^{15}N_\varepsilon$-$^{1}H_\varepsilon$ dipole interaction and $^{15}N_\varepsilon$ CSA are suppressed during the relaxation delay by application of proton 180° pulses at intervals of 100 ms, while cross-correlation effects between the $^{15}N_\varepsilon$-$^{13}C_{\delta,\zeta}$ dipole interactions and the $^{15}N_\varepsilon$ CSA and $^{13}C_\zeta$ CSA are neglected as justified above.

The transverse relaxation rate of $^{15}N_\varepsilon$ is obtained via the corresponding relaxation rate in the rotating frame. The direction of the effective field for the ^{15}N nucleus during the application of a spin-lock field, ω_{SL}, along $\hat{x}$ is $\hat{z}' = \sin(\theta)\hat{x} + \cos(\theta)\hat{z}$, where $\hat{x}$ and $\hat{z}$ are direction vectors in the rotating frame, $\tan(\theta) = \omega_{SL}/\Omega_N$, and Ω_N is the offset of the ^{15}N nucleus from the RF carrier. The relaxation rate of the anti-phase coherence in the rotating frame is then given by[9],

$$R_{1\rho}(2C_zN_z') = \cos^2(\theta)\, R_1(2C_zN_z) + \sin^2(\theta)\, R_2(2C_zN_x), \quad \text{(S9)}$$

which is measured with the scheme shown in Figure S2b. The pulse sequence elements before and after the spin-lock period in Figure S2b serve to align the magnetization with the effective spin-lock field, $\hat{z}'$, and return it to $\hat{z}$ as suggested previously.[13] Cross-correlations between the $^{15}N_\varepsilon$-$^{1}H_\varepsilon$ dipole interaction and $^{15}N_\varepsilon$ CSA are suppressed by application of a single proton 180° pulse in the middle of the spin-lock period.[14] The transverse anti-phase relaxation rate, $R_2(2C_zN_x)$, is subsequently calculated from the spin-lock field strength, ω_{SL}, the offset Ω_N, $R_1(2C_zN_z)$, and $R_{1\rho}(2C_zN_z')$ using Eq. S9. One advantage of obtaining the transverse relaxation rate via the relaxation rate in the rotating frame $R_{1\rho}(2C_zN_z')$ is that contributions from the exchange of $^{1}H_\varepsilon$ with the solvent are minimized.[15]

A pulse sequence to measure the longitudinal relaxation rate of $^{13}C_\zeta$ is shown in Figure S3. This sequence is very similar to the sequences shown in Figures 1 and S2, except that the decay of longitudinal in-phase $^{13}C_\zeta$ magnetization is encoded at the beginning of the sequence and is then followed by a $^{13}C_\zeta$-$^{15}N_\varepsilon$ HSQC (Figure 1). Although the obtained $R_1(C_z)$ rate is affected by $^{13}C_\zeta$-$^{1}H_{\varepsilon,\eta}$ dipole-dipole cross-correlation effects we show below that these rates are adequate for determining the in-phase $^{15}N_\varepsilon$ relaxation rates.

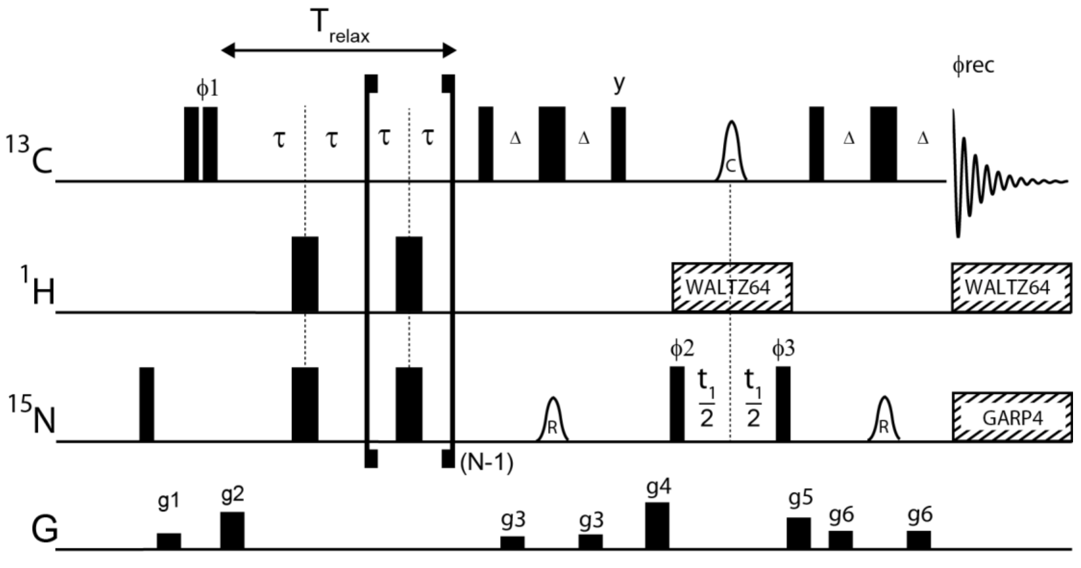

Figure S3: Pulse sequence for measuring $^{13}C_\zeta$ longitudinal relaxation. The carrier positions are ^{13}C: 159 ppm (100 ppm for decoupling using CHIRP pulse), ^{15}N: 84 ppm (78 ppm for decoupling) and ^1H: 7 ppm. 90° hard pulses are represented by narrow black rectangles, 180° pulses wide black rectangles and are applied at the highest possible power. The delay time $1/(4J_{C\zeta\text{-}N\varepsilon})$ =12.5 ms is depicted by the symbol Δ. Shaped pulses are represented by bell-shaped symbols, where letters inside the symbol specify the nature of the shaped pulse (R: RE-Burp, E: E-BURP-2[10], C: smoothed CHIRP[12]). Phases are *x* unless stated otherwise by ϕ indicating a phase cycle: ϕ_1: 4(*x*), 4(-*x*), ϕ_2: *x*, -*x* ϕ_3: 2(*x*), 2(-*x*), ϕ_{rec}: *x*, 2(-*x*), *x*, -*x*, 2(*x*), -*x*. Blocks of decoupling sequences are represented by boxes indicating the type of decoupling (WALTZ64[16] with a field strength of 4 kHz (carrier at 7 ppm) for proton decoupling and GARP4[17] with a field strength of 0.7 kHz (carrier at 78 ppm) for nitrogen decoupling). Smoothed-square gradients (1 ms) are represented by black rectangles and specified by a g*i* (g1: 9.5 G/cm, g2: 22.8 G/cm, g3: 3.9 G/cm, g4: 26.2 G/cm, g5: 18.4 G/cm, g6: 7.2 G/cm). Relaxation delays are $T_{relax} = 2 \cdot N \cdot \tau$, where N is an integer and τ=50 ms.

Overall, we derive the in-phase $^{15}N_\varepsilon$ relaxation rates from the anti-phase relaxation rates and $R_1(C_z)$ as described above (Eqs S1 and S2), and subsequently use these in-phase relaxation rates to derive motional parameters for the arginine side-chains.

Chemical Shift Assignments

For chemical shift assignments of the $^{13}C_\zeta$-$^{15}N_\varepsilon$ arginine side-chain resonances in proteins at neutral and high pH, we designed a 3D CCNeCz-TOCSY pulse sequence (see Figure S4) to correlate the aliphatic side-chain carbon chemical shifts with the $^{13}C_\zeta$-$^{15}N_\varepsilon$ resonances. We developed the pulse sequences for per-deuterated proteins dissolved in ^1H_2O or ^2H_2O since our goal is to probe arginine side-chains in large- to medium-sized proteins. A similar strategy with just minor modifications to the pulse scheme can be employed for studying protonated proteins. The $^{13}C_\zeta$-$^{15}N_\varepsilon$ resonances are assigned by comparing the aliphatic carbon chemical shifts from the 3D CCNeCz-TOCSY experiment

with those from a standard 3D CC(CO)NH-TOCSY[18] experiment (see below). Thus, matching aliphatic $^{13}C_{\alpha,\beta,\gamma,\delta}$ side-chain chemical shifts in the two experiments (Figure 2) links a given $^{13}C_{\zeta}$-$^{15}N_{\varepsilon}$ spin-pair to the corresponding ^{1}H-^{15}N backbone spin-pair of the following residue.

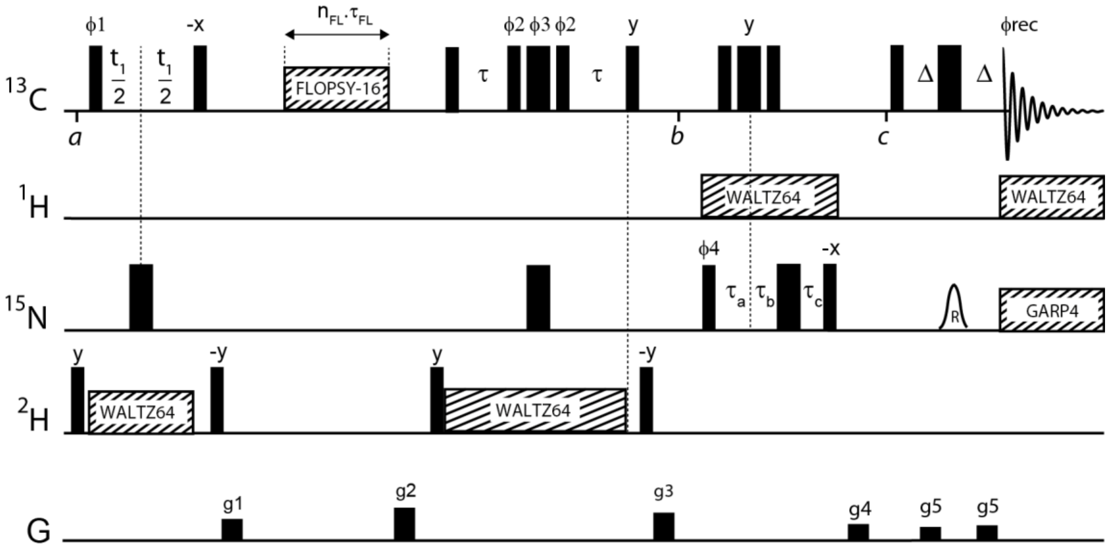

Figure S4: Pulse sequence for 3D CC-TOCSY-NeCz and assignment of $^{15}N_{\varepsilon}$ and $^{13}C_{\zeta}$ resonances. The carrier positions are ^{15}N: 84 ppm, ^{1}H: 7 ppm and ^{2}H: 3 ppm. The ^{13}C RF-carrier position is changed during the course of the pulse sequence, as depicted by the letters a, b and c (a: 43 ppm, b: 100 ppm, c: 159 ppm). 90° hard pulses are represented by narrow black rectangles, 180° pulses by wide black rectangles and are applied at the highest available power. The symbol Δ depicts the delay time 12.5 ms, $\tau = 1/(2J_{CC})$= 14.3 ms and τ_{FL}= 4.711 ms with n_{FL} = 4. A shaped RE-BURP pulse is represented by the bell-shaped symbol. Phases are *x* unless otherwise indicated; ϕ_1*: *x*, -*x*, ϕ_2: 4(*x*),4(-*x*) ϕ_3: 4(*y*), 4(-*y*), ϕ_4*: 2(*x*), 2(-*x*), ϕ_{rec}*: *x*, 2(-*x*), *x*, (*indicating States-TPPI acquisition). Blocks of decoupling sequences are represented by boxes indicating the type of decoupling, i.e., WALTZ64[16] with a field strength of 4 kHz (carrier at 7 ppm) for proton and 1 kHz (carrier at 3 ppm) deuterium decoupling and GARP4[17] with a field strength of 0.7 kHz (carrier at 78 ppm) for nitrogen decoupling. The FLOPSY-16 mixing sequence was applied at 10 kHz. Smoothed-square gradients (1 ms) are represented by black rectangles and specified by a g*i* (g1: 10.5 G/cm, g2: 23.8 G/cm, g3: 17.2 G/cm, g4: 7.2 G/cm, g5: 6.1 G/cm). The semi-constant time period has the following delays: $\tau_a = \Delta' + (n-1)(\Delta t_2/2)$, $\tau_b = (n-1)(\Delta t_2/2 - \Delta'/(N-1))$, $\tau_c = \Delta' - (n-1)(\Delta'/(N-1))$, where n = complex point index and N = total number of complex points, $\Delta t_2 = 1/sw(^{15}N_{\varepsilon})$ and Δ'= 15 ms.

Briefly, the 3D CCNeCz-TOCSY pulse sequence starts with excitation and indirect chemical shift evolution of aliphatic $^{13}C_{\alpha,\beta,\gamma,\delta}$ magnetization. The evolution time is restricted to 10 ms to limit carbon-carbon scalar coupling evolution. Following TOCSY mixing via the FLOPSY-16 scheme[19], $^{13}C_{\delta}$ magnetization is transferred to $^{15}N_{\varepsilon}$ via a constant-time period whose length is tuned to give zero net

$^{13}C_{\delta}$-$^{13}C_{\gamma}$ coupling evolution. The $^{15}N_{\varepsilon}$ chemical shift is encoded using a semi-constant-time[20] evolution period during which the $^{15}N_{\varepsilon}$-$^{13}C_{\delta}$ coupling is refocused. Concomitant evolution of the $^{15}N_{\varepsilon}$-$^{13}C_{\zeta}$ coupling leads to generation of magnetization that is anti-phase with respect to $^{13}C_{\zeta}$. Finally, this magnetization is transferred to in-phase C_{ζ} via a reverse-INEPT for detection. Deuterium, ^{2}H composite pulse decoupling (CPD) is applied when aliphatic carbon magnetization is transverse, ^{1}H CPD is applied during $^{15}N_{\varepsilon}$ chemical shift evolution and during acquisition, and ^{15}N CPD is applied during acquisition. This pattern of decoupling is applicable for side-chain deuterated samples in H_2O-based buffers. For samples in D_2O-based buffer, ^{2}H CPD is applied during ^{15}N chemical shift evolution, but not during acquisition.

In all instances, ^{2}H decoupling periods are flanked by 90° pulses to align the deuterium magnetization with the decoupling field, which minimizes perturbations to the ^{2}H lock signal. Arginine guanidino selectivity is ensured by using a $^{15}N_{\varepsilon}$ selective RE-BURP inversion pulse in the reverse-INEPT. Gradients are used to remove artifacts with frequency discrimination in both indirect dimensions achieved via States-TPPI.

Based on our success here with T4L L99A, where ten of the 13 arginine side-chains could be assigned using this method, we anticipate that the carbon-detected sequences are suitable for assignment of arginine side-chains of per-deuterated proteins up to ~20 kDa. Alternatively, the $^{13}C_{\zeta}$-$^{15}N_{\varepsilon}$ resonances can be assigned by site-directed mutagenesis, as shown below.

Experimental

Sample preparations

The T4 lysozyme mutant C54T/C97A/L99A (T4L L99A) was expressed in 1L M9 medium (2 g/L U-^{13}C glucose, 1 g/L $^{15}NH_4Cl$) at 18°C overnight and purified as described previously.[21] For the per-deuterated sample, cells were grown in D_2O and using deuterated $^{2}H_7$,$^{13}C_6$-glucose (Sigma-Aldrich). Samples were exchanged into NMR-buffer (50 mM sodium phosphate, 2 mM EDTA, 25 mM NaCl, 2 mM NaN_3, 10% D_2O, pH 5.5) and concentrated to a final concentration of *ca.* 0.8 mM - 2.5 mM.

The codon-optimized coding sequence of HDAC8 was obtained from GenScript (Piscataway, USA) in a pET-29b+ vector containing an N-terminal His-NusA-tag[22] separated from the HDAC8 coding sequence by a linker that contains a specific TEV cleavage site (ENLYFQG). The R223K-mutation was introduced by the Quikchange protocol. The wild-type and mutant constructs of HDAC8 were expressed in 2 or 4 L M9 medium (2.5-3 g/L U-^{13}C glucose, 1 g/L $^{15}NH_4Cl$) at 21°C overnight. Cells were harvested, re-suspended in lysis-buffer (50 mM Tris pH 8.0, 3 mM $MgCl_2$, 500

mM KCl, 5 mM β-mercaptoethanol, 5 mM imidazole, 5% glycerol, 0.25 % IGEPAL, 1 tablet of complete protease inhibitor (Roche) per 50 ml), traces of DNase I (Roche) and lysozyme (Sigma)), before lysis through sonication and subsequent centrifugation of the lysate at 25000 g for 45 mins. Purification over a first Ni-NTA column (GE Healthcare) was followed by dialysis into cleavage-buffer (50 mM Tris pH 8.0, 150 mM KCl, 5 mM β-mercaptoethanol, 5% glycerol) and cleavage by His-tagged TEV-protease. Cleaved HDAC8 was separated from the His-NusA-tag, the TEV-protease and non-specific contaminants by a passage through a second Ni-NTA column. The flow-through was pooled, concentrated and subjected to a gel filtration column (S75, GE-Healthcare) in gel-filtration buffer (50 mM Tris pH 8.0, 150 mM KCl, 1 mM TCEP, 5% glycerol). The protein was concentrated and buffer exchanged as specified in Table S1. Final sample concentrations were ~ 0.15-0.3 mM.

NMR experiments

All proton-detected backbone ^{15}N relaxation and 3D CC(CO)NH-TOCSY experiments were performed on a Bruker Avance III 500 MHz spectrometer using an HCN inverse room-temperature probe (TXI). Proton-detected side-chain ^{15}N relaxation experiments were recorded at both 500 MHz and 700 MHz. As confirmation and extension of our carbon-detected side-chain assignment we performed 3D (H)CC(CO)NH-TOCSY, 3D (H)CCNeHe-TOCSY and 3D HeNeCz assignment experiments (this approach is possible for T4 lysozyme as it is stable at pH 5.5), which were recorded on Varian Inova 600 MHz (14.1 T) and 800 MHz (18.8 T) spectrometers using HCN inverse coldprobes equipped with cooled ^{1}H preamplifiers (see Table S1 for further details).

For the carbon-detected side-chain relaxation experiments, the relaxation delays were 100, 200, 300, 400, 500 and 700 ms for $R_1(2C_zN_z)$ and $R_1(C_z)$ measurements and 2, 4, 8, 16, 32 and 64 ms for $R_{1\rho}(2C_zN_z')$ measurements, and other parameters were as described above.

Backbone ^{15}N relaxation rates (R_1 and $R_{1\rho}$) were measured at 500 MHz (11.74 T) using established proton-detected experiments[23] based on a gradient-selected, sensitivity-enhanced, refocused ^{15}N HSQC sequence.[24] The water signal was preserved using selective water pulses (2 ms sinc shape) and weak bipolar gradients were applied during the indirect chemical shift evolution to maintain the H_2O magnetization along *z*. In the $R_{1\rho}$ sequence, cross-correlation effects were suppressed by application of random-phase proton CW during the nitrogen spin-lock,[23] and magnetization was explicitly aligned with the spin-lock field.[13] Relaxation delays were 20, 200, 400, 700, 1000 and 1400 ms for R_1 measurements and 10, 20, 40, 60, 80 and 100 ms for $R_{1\rho}$ measurements. For the R_1 measurements, ^{15}N-^{1}H cross-correlation pathways were suppressed by application of amide-selective IBURP-1 pulses (1.92 ms at 500 MHz, centred at 8.27 ppm) at intervals of 10 ms during the relaxation delay.[10,25]

For measurement of the arginine $^{15}N_{\varepsilon}$ relaxation rates, the backbone ^{15}N R_1 and $R_{1\rho}$ relaxation sequences were modified by replacing the high-power ^{15}N 180° inversion pulse in the first INEPT element with a $^{15}N_{\varepsilon}$-selective RE-BURP pulse[10] (3.4 ms at 700 MHz). $^{15}N_{\varepsilon}$-$^{1}H_{\varepsilon}$ cross-correlation during the relaxation delay of the $R_{1\rho}$ experiment recorded at 700 MHz was suppressed using a single $^{1}H_{\varepsilon}$-selective IBURP-1 pulse in the middle of the spin-lock period. Relaxation delays were 100, 200, 300, 400, 500 and 700 ms for R_1 measurements and 2, 4, 8, 16, 32 and 64 ms for $R_{1\rho}$ measurements. Side-chain {^{1}H}-^{15}N steady-state heteronuclear NOEs were measured using standard methods.[24] The high power ^{15}N excitation pulse was replaced with a N_{ε}-selective E-BURP-2 pulse (2.57 ms at 700 MHz). Water magnetization was preserved in the reference spectrum as described above. Saturation of proton magnetization was achieved using a 5 s train of high-power 120° pulses applied at 5 ms intervals. The reference and saturated spectra were recorded in an interleaved fashion, and therefore to ensure full recovery of the water magnetization at the start of each increment of the reference experiment, a long recycle delay of 15 s was used.

For the comparison of signal/noise ratios between the proton-detected and the carbon-detected HSQC of T4 lysozyme L99A, both spectra were processed with the same shifted square sine window function. Noise levels and peak intensities were determined by NMRpipe[26] to calculate an average signal-to-noise ratio for each spectrum, which was further normalized by the acquisition time of the experiment.

A comprehensive list of all experiments including sample details, experimental conditions, and recording parameters is provided separately (see Table S1).

Calculation of order parameters

Rotating frame $R_{1\rho}$ relaxation rates were converted to R_2 relaxation rates using Eq. S9. R_2/R_1 ratios were calculated for those backbone amides that have limited flexibility and minimal chemical exchange.[27] Subsequently the R_2/R_1 ratios were used as inputs to calculate the overall diffusion tensor, **D**, and local correlation times ($\tau_{R,local}$) of specific arginine backbone ^{1}H-^{15}N and side-chain $^{1}H_{\varepsilon}$-$^{15}N_{\varepsilon}$ bond-vectors of T4L L99A (PDB: 3dmv[28] with protons added) using the program *quadric_diffusion*[29] and assuming an axially symmetric diffusion tensor.

Order parameters were calculated from $^{15}N_{\varepsilon}$ side-chain R_2 and R_1 relaxation rates (from carbon detected and proton detected side-chain experiments) by using the calculated τ_R as a constraint and solving Eqs S3, S4, and S8 numerically for S^2 and τ_e' using the Octave numerical software package (http://www.gnu.org/software/octave/).

Side-chain order parameters were also calculated by including heteronuclear NOEs measured by proton-detected experiments. With this additional dataset order parameters were calculated by a χ^2 minimization in Octave using Eqs. S3, S4, S5 and S8, again constraining τ_R as above. These order parameters obtained from three parameters (R_1, R_2, NOE) were compared to those obtained from two parameters (R_1, R_2) and showed good agreement (see below, Figure S9). The propagation of errors was calculated by Monte Carlo simulation[30] using at least 10 randomly generated and normally distributed datasets. The error for the input parameters (the standard deviation of the randomly generated datasets) was set to the experimentally determined error or 2% of the parameter value, whichever was the largest. In the case of the NOE data the uncertainty was set to 2% of the highest parameter value.

Assignment of R223 and potassium titrations

The $^{13}C_{\zeta}$-$^{15}N_{\varepsilon}$ correlation spectra of the HDAC8 mutant R223K were recorded in the same potassium-phosphate buffer as the wild-type (see Table S1) and spectra of wild type and mutant were overlaid (see Figure 3). HDAC8 has been proposed to be regulated allosterically by potassium ions[31] and consequently mutations might cause perturbations in other parts of the enzyme. No other arginines are in the close vicinity of R223, the closest arginine to R223 in the crystal structure (PDB code: 2V5W) being approximately 20 Å away. We therefore conclude that the obvious disappearance of a dispersed peak as shown in Figure 3 is due to the absence of an arginine residue at position 223 and attribute slight changes of the intensities seen in the random-coil region of the spectrum to secondary effects caused by the mutation.

Potassium titrations of HDAC8 were performed in Tris-buffer (25 mM Tris pH 8.0, 0.5 mM TCEP, 1 mM NaN3, 0.001% DSS and 10% D2O) with 1, 10, 100 and 200 mM KCl and approximately 0.2 mM HDAC8. Two-dimensional $^{13}C_{\zeta}$-$^{15}N_{\varepsilon}$ HSQC spectra were recorded using the sequence presented in Figure 1. The experimental time for one titration point was approximately 19 h. The R223 cross-peak was hardly visible at low concentrations of potassium and its intensity increases as the concentration of potassium is increased. Thus, the binding/release of the potassium ion is in the slow exchange regime[32], i.e. on the order of or slower than ~10 ms, since a change in intensity of the R223 peak is observed rather than a change in peak position. The fact that we could not observe an isolated peak disappearing in the $^{13}C_{\zeta}$-$^{15}N_{\varepsilon}$ HSQC spectrum as the concentration of potassium was increased suggests that the arginine side-chain of the potassium-free state of HDAC8 is either disordered or undergoing millisecond chemical exchange such that the corresponding resonance either becomes masked in the random-coil region of the spectrum or broadened beyond detection, respectively. Peak volumes and errors were extracted from a fit of Gaussian line-shape as implemented in Sparky.[33] Furthermore, these were corrected for differences in protein concentration as estimated

from the relative area of the methyl regions in 1D ^{1}H-detected spectra. To correct for the loss of sensitivity due to higher ionic strength, the proton signals of the Tris-buffer, which is constant in concentration during the titration, were used as references. Subsequently, the obtained normalized intensities were fitted to a hyperbolic binding curve,

$$I([K^{+}]) = I_0 + I_{max}(1+K_D/[K^{+}])^{-1} \tag{S10}$$

where $I([K^{+}])$ is the corrected peak volume at a given potassium concentration $[K^{+}]$, I_0 and I_{max} the (corrected) peak volumes at zero and saturating potassium concentrations, respectively, and K_D the dissociation constant of potassium binding. The data in Figure 3d are normalized such that I_{max} = 1.0.

Experimental validations using T4 lysozyme L99A

Isotope shifts in the $^{13}C_{\zeta}$-$^{15}N_{\varepsilon}$ spectrum

The peak shape in the $^{13}C_{\zeta}$-$^{15}N_{\varepsilon}$ HSQC spectrum of T4 L99A (Figure 2b) is not fully symmetrical and for the intense signals we observe some residual upfield-shifted peaks (see for example at 159.5 ppm and 83.6 ppm in Figure 2b). These weak residual peaks are caused by the 10% D_2O in the samples that results in a distribution of isotopomers and consequently isotope shifts.[34] In this study we recorded the spectrum in 90% H_2O/10% D_2O to allow comparison of the motional parameters derived from the carbon-detected experiments with those derived from conventional proton-detected experiments. For applications at neutral-to-high pH, a 100% $^{2}H_2O$ buffer could often be preferable. For comparison, Figure S5 shows a spectrum of T4L L99A in ~100% D_2O. In our study, however, the small residual isotopomer peaks did not hamper either the chemical shift assignment or the relaxation measurements of T4L L99A.

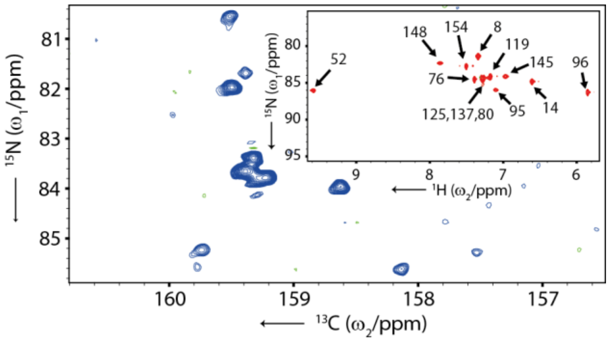

Figure S5: $^{13}C_{\zeta}$-$^{15}N_{\varepsilon}$-HSQC of T4 lysozyme L99A in ~ 100% D_2O buffer recorded at a magnetic field of 16.4 T and a temperature of 298 K. The inset shows the corresponding $^{1}H_{\varepsilon}$-$^{15}N_{\varepsilon}$ HSQC spectrum recorded in 10% D_2O buffer.

Chemical shift dispersion of the $^{13}C_\zeta$-$^{15}N_\varepsilon$ spectrum

The $^{13}C_\zeta$-$^{15}N_\varepsilon$ and $^{1}H_\varepsilon$-$^{15}N_\varepsilon$ spectra show similar levels of chemical shift dispersion (Figure S5) indicating the feasibility of the carbon-detected method for probing individual arginine side-chains. As shown in Figure S6 below, the numbers of expected overlapped peaks in the two spectra, $^{13}C_\zeta$-$^{15}N_\varepsilon$ and $^{1}H_\varepsilon$-$^{15}N_\varepsilon$ HSQC, are very similar. Briefly, we randomly generated two-dimensional spectra using peak positions published in the BMRB database[35] and linewidths extracted from the spectra in Figure 2b and Figure S5. For different numbers of peaks we calculated the most probable number of overlaps and found that the number of predicted overlaps is very similar for the $^{13}C_\zeta$-$^{15}N_\varepsilon$ and $^{1}H_\varepsilon$-$^{15}N_\varepsilon$ spectra. For example, for a protein with 13 arginines, there will be on average 0.95±1.1 overlaps of peaks in the $^{1}H_\varepsilon$-$^{15}N_\varepsilon$ spectrum, while 1.05 ± 1.1 overlaps are expected in the $^{13}C_\zeta$-$^{15}N_\varepsilon$ spectrum. As much as these simulations might be biased by the peak positions published in the BMRB database, they demonstrate that a similar overlap of peaks is expected in the two spectra.

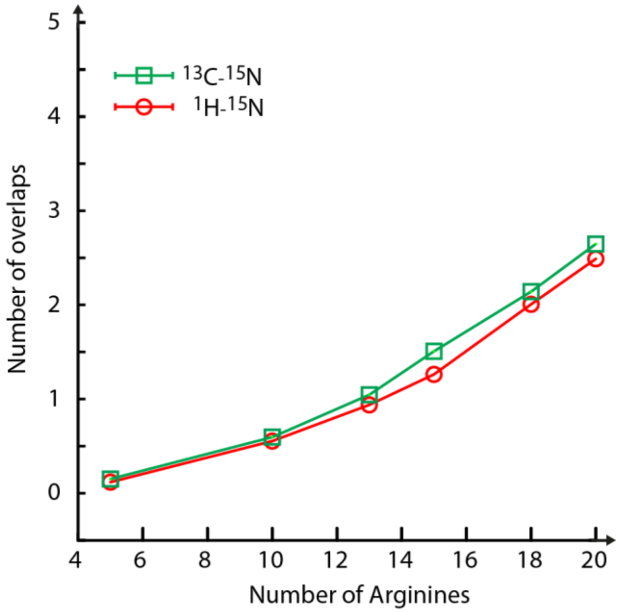

Figure S6: Predicted number of overlaps in the $^{13}C_\zeta$-$^{15}N_\varepsilon$ (green) and $^{1}H_\varepsilon$-$^{15}N_\varepsilon$ (red) HSQC spectra, respectively. The predicted overlaps are calculated from simulations where {5,10,13,15,18,20} arginine side-chain chemical shifts were taken randomly from the BMRB database.[35] Linewidths used for the simulation of the $^{13}C_\zeta$, $^{15}N_\varepsilon$ and $^{1}H_\varepsilon$ resonances are calculated from a Gaussian distribution (mean ± width) of those measured in the $^{13}C_\zeta$-$^{15}N_\varepsilon$ spectrum of T4L L99A shown in Figure 2b and a $^{1}H_\varepsilon$-$^{15}N_\varepsilon$ HSQC spectrum. For simulations of $^{13}C_\zeta$-$^{15}N_\varepsilon$ spectra, linewidths of $\Delta\nu_{1/2}(^{13}C_\zeta)$ = 7.9±4.0 Hz and $\Delta\nu_{1/2}(^{15}N_\varepsilon)$ = 5.9±2.7 Hz were used, while for simulations of the $^{1}H_\varepsilon$-$^{15}N_\varepsilon$ spectra, $\Delta\nu_{1/2}(^{1}H_\varepsilon)$ = 15.1±9.7 Hz and $\Delta\nu_{1/2}(^{15}N_\varepsilon)$ = 12.5±3.0 Hz were used. Spectra were simulated and the number of overlaps counted according to when two ellipses, defined by the peak positions and their linewidths, intersected. The numbers shown in the figure are averages of 10 000 simulations.

Consistencies of derived relaxation rates from carbon-detected and proton-detected spectra.

As described above, we used $^{13}C_{\zeta}$-$^{15}N_{\varepsilon}$ correlated spectra to determine the $^{15}N_{\varepsilon}$ relaxation rates and subsequently derive motional parameters for arginine side-chains. More specifically, the anti-phase spin relaxation rates $R_1(2C_zN_z)$ and $R_{1\rho}(2C_zN'_z)$ were measured for the peaks shown in Figure 2b. In an independent experiment, we measured $R_1(C_z)$ in order to calculate the pure in-phase $^{15}N_{\varepsilon}$ relaxation rates, $R_1(N_z)$ and $R_{1\rho}(N_z')$, according to Eqs S1 and S2. As an initial validation of our strategy to quantify molecular motions of arginine side-chains using carbon-detected experiments, we compared these rates obtained from the $^{13}C_{\zeta}$-$^{15}N_{\varepsilon}$-type experiments with the corresponding $R_1(N_z)$ and $R_{1\rho}(N_z')$ rates obtained using standard proton-detected experiments.[25] This comparison is shown in Figure S7 where the $^{15}N_{\varepsilon}$ longitudinal relaxation rate, $R_1(N_z)$, and relaxation rate in the rotating frame, $R_{1\rho}(N_z')$, derived from the $R_1(2C_zN_z)$, $R_{1\rho}(2C_zN_z')$ and $R_1(C_z)$ rates are seen to agree well with the corresponding rates obtained from $^{1}H_{\varepsilon}$-$^{15}N_{\varepsilon}$-type experiments. For the seven isolated peaks in Figure 2b (R8, R14, R52, R95, R96, R148, R154), we obtain RMSD($R_{1,CN}$, $R_{1,HN}$) = 0.14 s^{-1} and RMSD($R_{1\rho,CN}$, $R_{1\rho,HN}$) = 0.71 s^{-1}. Including the peaks of the more crowded region (shown with green labels in Figure 2b) gives the same general picture, despite the higher uncertainty in these additional relaxation rate constants.

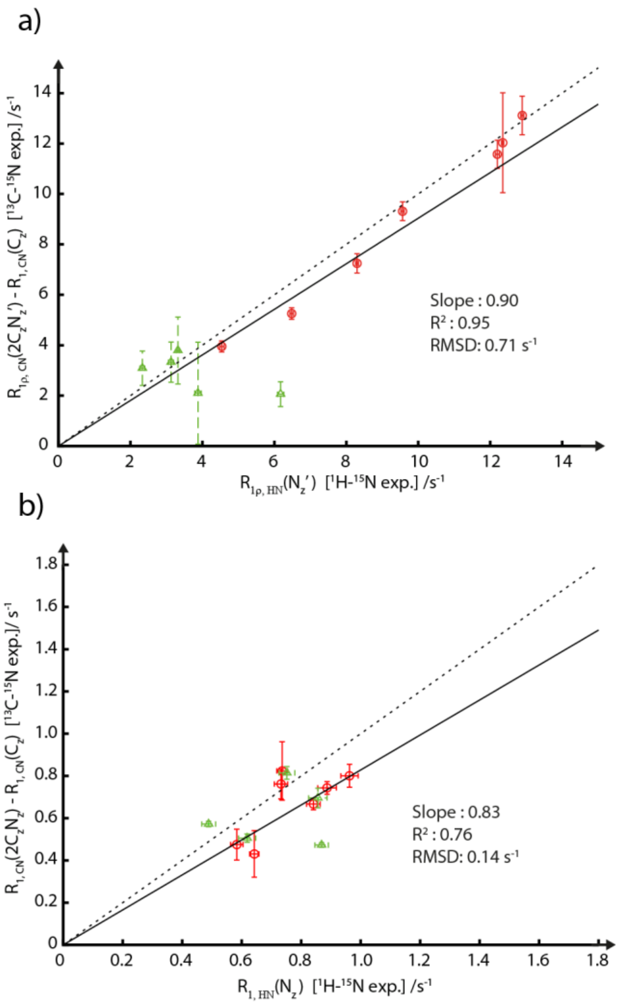

Figure S7: Correlation of (a) $^{15}N_{\varepsilon}$ $R_{1\rho}$ and (b) $^{15}N_{\varepsilon}$ R_1 relaxation rates obtained from carbon-detected and proton detected experiments. (a) Comparison of the $^{15}N_{\varepsilon}$ $R_{1\rho}$ relaxation rates extracted from carbon-detected $^{13}C_{\zeta}$-$^{15}N_{\varepsilon}$-type experiments, [^{13}C-^{15}N exp.], and conventional proton-detected $^{1}H_{\varepsilon}$-$^{15}N_{\varepsilon}$-type experiments, [^{1}H-^{15}N exp.]. Uncertainties in $R_{1\rho}$ were calculated from standard errors from least-squares fits. Red circles refer to data points obtained from fitting relaxation data of the disperse peaks (R8, R14, R52, R95, R96, R148, R154), while green triangles refer to data from peaks in the crowded region of the spectrum, that is R76, R80, R119, R125, R137 (see Figure 2b). The solid line represents a fit (*y=ax*) of the red data points with a slope of 0.90 ± 0.03, a squared Pearson coefficient of linear correlation (R^2) of 0.95 and RMSD($R_{1\rho,CN}$, $R_{1\rho,HN}$) = 0.71 s^{-1}. The dashed line represents *y=x*. (b) Longitudinal relaxation rate constants, $R_1(N_z)$, obtained for T4L L99A from the $^{13}C_{\zeta}$-$^{15}N_{\varepsilon}$-based experiments described in Figure 1 and S1 are plotted on the *y*-axis, and the *x*-axis represents the corresponding rate constant obtained from experiments based on proton excitation and detection. Error bars represent standard errors of the fitted parameters. Symbols and colours are the same as (a). The solid line represents a fit (*y=ax*) of the red data points with a slope of 0.83 ± 0.03. The dashed line represents *y=x*.

Whereas $^{15}N_{\varepsilon}$ R_2 rates in Figure S7 cover a wide range of values (from approximately 2 s^{-1} to 13 s^{-1}), the $^{15}N_{\varepsilon}$ R_1 rates cover a much narrower range (approximately 0.4 s^{-1} – 0.9 s^{-1}). In Figure S8 we

show that this is the expected outcome for arginine side-chains with local motions on time-scales from $\tau_e' \approx 0.1 - 0.5$ ns (see Equation S8).

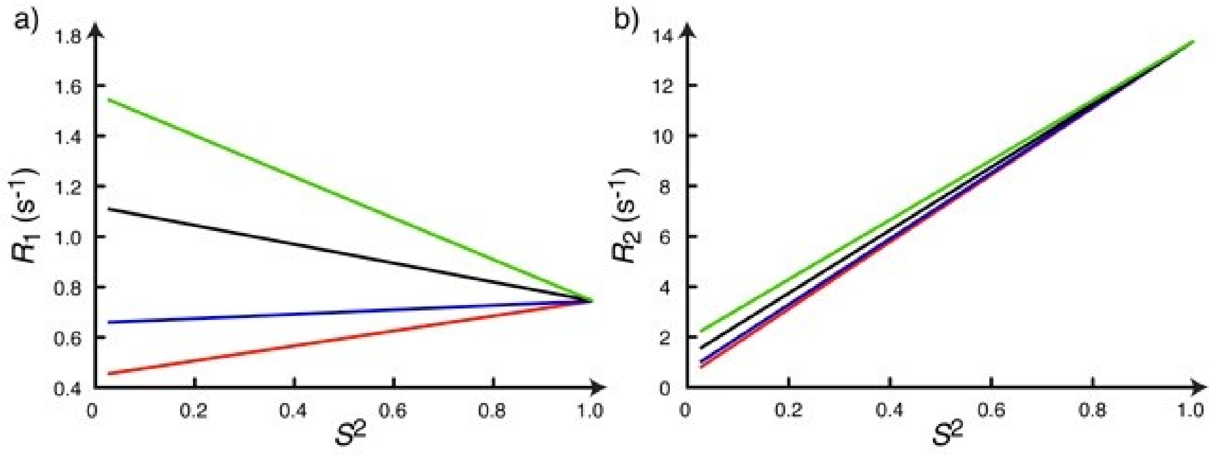

Figure S8: Simulated relaxation rates a) R_1 and b) R_2 for a 16.4 T static magnetic field using a $^{15}N_\varepsilon$ CSA of –114 ppm, a N-H bond-length of 1.02 Å, and an overall rotational correlation time of 11.6 ns, which was determined experimentally from backbone ^{15}N relaxation rates of T4L L99A. Four local correlation times, τ_e, were used: 1 ns (green), 0.5 ns (black), 0.1 ns (red), and 0.169 ns (blue; average local correlation time for the arginine side-chains of T4L L99A).

In Figure 2d we calculate the side-chain order parameters solely from the R_1 and R_2 rates, whereas order parameters are often calculated from the three rates, R_1, R_2, and $\{^1H\}$-^{15}N NOEs. Figure S9 shows that arginine side-chain order parameters calculated solely from the R_1 and R_2 rates are in good agreement with the corresponding order parameters calculated from the three rates, thus justifying our approach.

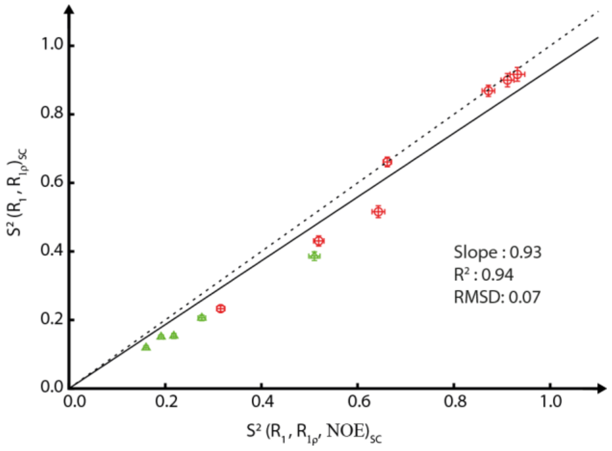

Figure S9: Comparison of T4L L99A arginine $^{15}N_\varepsilon$-$^1H_\varepsilon$ order parameters determined by two different approaches both using the same proton-detected raw data. Order parameters that were calculated using R_1, R_2 and $\{^1H\}$-^{15}N NOE rates (proton-detected experiments) are plotted on the *x*-axis, while order parameters calculated without NOE data are plotted on the *y*-axis. Symbols and colour code are as described in Figure 2d.

Comparison of side-chain and backbone order parameters

The derived order parameters for the arginine side-chains of T4L L99A range from approximately 0.1 to 0.9 showing that some arginine side-chains (S^2 ~ 0.1) are nearly completely uncoupled from the overall tumbling while other arginine side-chains (S^2 ~ 0.9) are as rigid as the backbone. This is in agreement with previous results obtained for methyl-bearing side-chains, where the range of order parameters correlates with the number of degrees of freedom for the side-chain.[36] The fact that we see this wide range of order parameters for the arginine side-chain is therefore in agreement with possible motions around the four side-chain dihedral angles (χ_1, χ_2, χ_3, χ_4).

In general, the side-chains of amino acids probe a different environment from that of the backbone. We have compared the side-chain order parameters, S^2, of T4L L99A with those derived from the backbone. This comparison is shown in Figure S10. Although the results confirm earlier studies[2,37] in showing that the side-chain and the backbone motions are largely uncoupled, it once again underlines the need to probe the side-chain moieties specifically in order to gain insight into their dynamics.

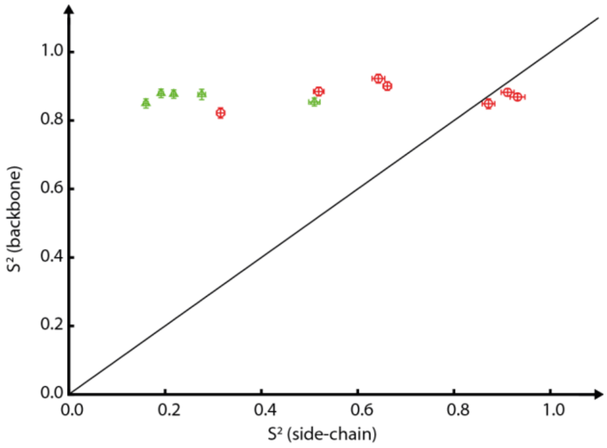

Figure S10: **Comparison of arginine side-chain and backbone order parameters.** Comparison of arginine $^{15}N_\varepsilon$-$^{1}H_\varepsilon$ order parameters with backbone ^{15}N-$^{1}H_N$ order parameters for T4L L99A. ^{15}N-^{1}H order parameters for the arginine side-chains of T4L L99A (*x*-axis) and backbone (*y*-axis) were determined from the proton-detected experiments. The R_2 rates were not corrected for the contribution from chemical exchange, $R_{ex,}$, because the R_{ex} contributions calculated under the applied conditions[27,38] were in general small (<0.5 s^{-1}, < 3 %). The contribution from chemical exchange was suppressed, because the $R_{1\rho}$ rates used to calculate R_2 were measured using a spin-lock field (ω_{SL} = 2π ·1500 rad/s) that is larger than the chemical exchange rate (k_{ex} ~ 1000 s^{-1}).[38] The solid line represents *y*=*x*. It is clear that the side-chain order parameters and thus dynamics of the side-chains are very different from the backbone dynamics, as has also been observed for other side-chains.

Experiment	Pulse-Sequence	Sample	Labelling	Concentration	Buffer	Spectrometer	$t_{1,max}$	NS	Measurement time	Data presented/ included
$^{13}C_{\zeta}$-$^{15}N_{\varepsilon}$ HSQC	Figure 1	T4L L99A	13C-15N-2H	2.5 mM	A	16.4 T	100 ms	8	0h30min	Figure 2B
$^{13}C_{\zeta}$-$^{15}N_{\varepsilon}$ HSQC	Figure 1	T4L L99A	13C-15N	0.8 mM	A(D_2O)	16.4 T	60.0 ms	16	0h55min	Figure S5
^{1}H-^{15}N-HSQC		T4 L99A	13C-15N-2H	2.5 mM	A	16.4 T	78.3 ms	4	1h1min	Figure S5 inset
CC(CO)NH-TOCSY	[18]	T4 L99A	13C-15N-2H	2.5 mM	A	11.7 T	23.6 ms (^{15}N)<br>8.0 ms (^{13}C)	16	2d22h	Figure 2B-C
CCNeCz-TOCSY	Figure S4	T4 L99A	13C-15N-2H	2.5 mM	A	16.4 T	64.0 ms (^{15}N)<br>8.8 ms (^{13}C)	8	2d19h	Figure 2B-C
(H)CC(CO)NH-TOCSY	[18]	T4 L99A	13C-15N	1.4 mM	A	18.8 T	16 ms (^{15}N)<br>7 ms (^{13}C)	8	2d4h	Figure 2B
H(C)C(CO)NH-TOCSY	[18]	T4 L99A	13C-15N	1.4 mM	A	18.8 T	17 ms (^{15}N)<br>14 ms (^{1}H)	8	2d4h	
(H)CCNH-TOCSY	[18c, 39]	T4 L99A	13C-15N	1.4 mM	A	14.1 T	22 ms (^{15}N)<br>7 ms (^{13}C)	8	1d6h	Figure 2B
H(C)CNH-TOCSY	[18c, 39]	T4 L99A	13C-15N	1.4 mM	A	14.1 T	22 ms (^{15}N)<br>10 ms (^{1}H)	8	1d6h	
HNeCz	[40]	T4 L99A	13C-15N	1.4 mM	A	14.1 T	24.1 ms (^{15}N)<br>17.8 ms (^{13}C)	4	1d6h	Figure 2B
$^{13}C_{\zeta}$-$^{15}N_{\varepsilon}$ $R_1(2C_zN_z)$	Figure S2	T4 L99A	13C-15N-2H	2.5 mM	A	16.4 T	76.8 ms	96	2d10h	Figure 2d, S7b
$^{13}C_{\zeta}$-$^{15}N_{\varepsilon}$ $R_{1\rho}(2C_zN_z')$	Figure S2	T4 L99A	13C-15N-2H	2.5 mM	A	16.4 T	76.8 ms	96	2d17h	Figure 2d, S7a
$^{1}H_{\varepsilon}$-$^{15}N_{\varepsilon}$ $R_1(N_z)$	[24]	T4 L99A	13C-15N-2H	2.5 mM	A	16.4 T	53.2 ms	8	1d10h	Figure 2d, S7b
$^{1}H_{\varepsilon}$-$^{15}N_{\varepsilon}$ $R_{1\rho}(N_z')$	[23,24]	T4 L99A	13C-15N-2H	2.5 mM	A	16.4 T	53.2 ms	16	1d7h	Figure 2d, S7a
$^{1}H_{\varepsilon}$-$^{15}N_{\varepsilon}$ $R_1(N_z)$	[24]	T4 L99A	13C-15N-2H	2.5 mM	A	11.7 T	47.7 ms	8	19h23min	Figure S9, S10
$^{1}H_{\varepsilon}$-$^{15}N_{\varepsilon}$ $R_{1\rho}(N_z')$	[23,24]	T4 L99A	13C-15N-2H	2.5 mM	A	11.7 T	47.7 ms	16	1d15h	Figure S9, S10
{$^{1}H_{\varepsilon}$}-$^{15}N_{\varepsilon}$ NOE	[24, 41]	T4 L99A	13C-15N-2H	2.5 mM	A	11.7 T	98.3 ms	8	1d12h	Figure S9, S10
^{1}H-^{15}N $R_1(N_z)$	[24]	T4 L99A	13C-15N-2H	2.5 mM	A	11.7 T	94.3 ms	8	19h29min	Figure 2d, S9, S10
^{1}H-^{15}N $R_{1\rho}(N_z')$	[23,24]	T4 L99A	13C-15N-2H	2.5 mM	A	11.7 T	94.3 ms	16	1d14h	Figure 2d, S9, S10
{^{1}H}-^{15}N NOE	[24, 41]	T4 L99A	13C-15N-2H	2.5 mM	A	11.7 T	377.1 ms	8	2d10h	Figure S10
$^{13}C_{\zeta}$-$^{15}N_{\varepsilon}$ $R_1(C_z)$	Figure S3	T4 L99A	13C-15N-2H	2.5 mM	A	16.4 T	76.8 ms	80	1d20h	Figure 2d, S7
$^{13}C_{\zeta}$-$^{15}N_{\varepsilon}$ -HSQC	Figure 1	HDAC8	13C-15N	0.4 mM	B	16.4 T	22. 4 ms	320	14h53min	Figure 3

^{1}H-^{15}N-TROSY	[42]	HDAC8	13C-15N	0.3 mM	B	16.4 T	50.1 ms	64	10h35min	Figure 3
$^{13}C_{\zeta}$-$^{15}N_{\varepsilon}$ -HSQC	Figure 1	HDAC8-R223K	13C-15N	0.2 mM	B	16.4 T	22. 4 ms	320	14h53min	Figure 3
K^{+}-titration: $^{13}C_{\zeta}$-$^{15}N_{\varepsilon}$ -HSQC	Figure 1	HDAC8	13C-15N	~0.2 mM	C	16.4 T	22. 4 ms	512	19h5min	Figure 3

Table S1: Experimental parameters, samples and experimental conditions for data described in main text and supporting material. *Sample* refers to protein samples as specified in the main text, their isotope labeling schemes and concentrations. The sample buffers are as follows: A: 50 mM sodium phosphate, 2 mM EDTA, 25 mM NaCl, 2 mM NaN_3, 10% D_2O, pH 5.5, B: 50 mM potassium phosphate, 30 mM KCl, 1 mM DTT, 1mM NaN_3 in 100% D_2O at pH 8.2, C: 25 mM Tris pH 8.0, 0.5 mM TCEP, 1 mM NaN_3, 0.001% DSS and 10% D_2O with 1, 10, 100, 200 mM KCl or 1mM KCl and 100 mM NaCl (see main text for details). *Spectrometer* specifies the field strength (in tesla) of the spectrometers used as described in the Materials and Methods section, $t_{1,max}$ is the acquisition time in the indirect dimension (or both indirect dimensions for 3D experiments), *NS* the number of scans and *Measurement time* the total experimental time. The last column indicates where the respective experiment has been included.

References

[1] A. G. Palmer, *Annu. Rev. Biophys. Biomol. Struct.* **2001**, *30*, 129–55.

[2] N. Trbovic, J.-H. Cho, R. Abel, R. A. Friesner, M. Rance, A. G. Palmer, *J. Am. Chem. Soc.* **2009**, *131*, 615–22.

[3] M. Tang, A.-J. Waring, M. Hong, *ChemBioChem* **2008**, *9*, 1487–92.

[4] P. Allard, M. Helgstrand, T. Hard, *J. Magn. Reson.* **1998**, *134*, 7–16.

[5] D. F. Hansen, P. Vallurupalli, P. Lundström, P. Neudecker, L. E. Kay, *J. Am. Chem. Soc.* **2008**, *130*, 2667–75.

[6] (a) S. Kawato, K. Kinosita, A. Ikegami, *Biochemistry* **1977**, *16*, 2319–2324. (b) Woessner, D. E. *J. Chem. Phys.* **1965**, *42*, 1855.

[7] (a) G. Lipari, A. Szabo, *J. Am. Chem. Soc.* **1982**, *104*, 4546–4559. (b) Lipari, G.; Szabo, A. *J. Am. Chem. Soc.* **1982**, *104*, 4559–4570.

[8] (a) A. Abragam, *Principles of Nuclear Magnetism*; Clarendon Press: Oxford, 1961. (b) J. W. Peng, G. Wagner, *Methods Enzymol.* **1994**, *239*, 563–96.

[9] (a) N. A. Farrow, O. Zhang, A. Szabo, D. A. Torchia, L. E. Kay, *J. Biomol. NMR* **1995**, *6*, 153–62. (b) J. W. Peng, G. Wagner, *J. Magn. Reson.* **1992**, *98*, 308–332. (c) R. Ishima, K. Nagayama, *Biochemistry* **1995**, *34*, 3162–71.

[10] H. Geen, R. Freeman, *J. Magn. Reson.* **1991**, *93*, 93–141.

[11] I. André, S. Linse, F. A. A. Mulder, *J. Am. Chem. Soc.* **2007**, *129*, 15805–13.

[12] J. M. Bohlen, G. Bodenhausen, *J. Magn. Reson., Ser. A* **1993**, *102*, 293–301.

[13] D. F. Hansen, L. E. Kay, *J. Biomol. NMR* **2007**, *37*, 245–55.

[14] D. F. Hansen, D. Yang, H. Feng, Z. Zhou, S. Wiesner, Y. Bai, L. E. Kay, *J. Am. Chem. Soc.* **2007**, *129*, 11468–79.

[15] T. Segawa, F. Kateb, L. Duma, G. Bodenhausen, P. Pelupessy, *ChemBioChem* **2008**, *9*, 537–42.

[16] (a) A. J. Shaka, J. Keeler, T. Frenkiel, R. Freeman, *J. Magn. Reson.* **1983**, *52*, 335–338. (b) Z. Zhou, R. Kümmerle, X. Qiu, D. Redwine, R. Cong, A. Taha, D. Baugh, B. Winniford, *J. Magn. Reson.* **2007**, *187*, 225–33.

[17] A. J. Shaka, P. B. Barker, R. Freeman, *J. Magn. Reson.* **1985**, *64*, 547–552.

[18] (a) G. T. Montelione, B. A. Lyons, S. D. Emerson, M. Tashiro, *J. Am. Chem. Soc.* **1992**, *114*, 10974–10975. (b) S. Grzesiek, J. Anglister, A. Bax, *J. Magn. Reson., Ser. B* **1993**,

101, 114–119. (c) T. M. Logan, E. T. Olejniczak, R. X. Xu, S. W. Fesik, *FEBS Lett.* **1992**, *314*, 413–8.

[19] M. Kadkhodaie, O. Rivas, M. Tan, A. Mohebbi, A. Shaka, *J. Magn. Reson.* **1991**, *91*, 437–443.

[20] (a) T. M. Logan, E. T. Olejniczak, R. X. Xu, S. W. Fesik, *J. Biomol. NMR* **1993**, *3*, 225–31. (b) S. Grzesiek, A. Bax, *J. Biomol. NMR* **1993**, *3*, 185–204.

[21] P. Vallurupalli, D. F. Hansen, P. Lundström, L. E. Kay, *J. Biomol. NMR* **2009**, *45*, 45–55.

[22] V. De Marco,G. Stier, S. Blandin, A. de Marco, *Biochem. Biophys. Res. Commun.* **2004**, *322*, 766–71.

[23] D. M. Korzhnev, N. R. Skrynnikov, O. Millet, D. A. Torchia, L. E. Kay, *J. Am. Chem. Soc.* **2002**, *124*, 10743–53.

[24] (a) L. E. Kay, D. A. Torchia, A. Bax, *Biochemistry* **1989**, *28*, 8972–8979. (b) K. T. Dayie, G. Wagner, *J. Magn. Reson., Ser. A* **1994**, *111*, 121–126.

[25] (a) N. A. Farrow, R. Muhandiram, A. U. Singer, S. M. Pascal, C. M. Kay, G. Gish, S. E. Shoelson, T. Pawson, J. D. Forman-Kay, L. E. Kay, *Biochemistry* **1994**, *33*, 5984–6003. (b) L. E. Kay, L. K. Nicholson, F. Delaglio, A. Bax, D. A. Torchia, *J. Magn. Reson.* **1992**, *97*, 359–375.

[26] F. Delaglio, S. Grzesiek, G. W. Vuister, G. Zhu, J. Pfeifer, A. Bax, *J. Biomol. NMR* **1995**, *6*, 277–93.

[27] G. Bouvignies, P. Vallurupalli, D. F. Hansen, B. E. Correia, O. Lange, A. Bah, R. M. Vernon, F. W. Dahlquist, D. Baker, L. E. Kay, *Nature* **2011**, *477*, 111–4.

[28] L. Liu, W. A. Baase, B. W. Matthews, *J. Mol. Biol.* **2009**, *385*, 595–605.

[29] L. K. Lee, M. Rance, W. J. Chazin, A. G. Palmer, *J. Biomol. NMR* **1997**, *9*, 287–98.

[30] W. H. Press, S. A. Teukolsky,W. T. Vetterling, B. P. Flannery, *Numerical Recipes in C++: The Art of Scientific Computing*; Cambridge University Press.

[31] S. L. Gantt, C. G. Joseph, C. A. Fierke, *J. Biol. Chem.* **2010**, *285*, 6036–43.

[32] H. M. McConnell, *J. Chem. Phys.* **1958**, *28*, 430–31.

[33] T. D. Kneller, D. G. Goddard, SPARKY 3

[34] (a) J. Abildgaard, P. E. Hansen, M. N. Manalo, A. LiWang, *J. Biomol. NMR* **2009**, *44*, 119–26. (b) P. E. Hansen, *Magn. Reson. Chem.* **2008**, *46*, 726–9.

[35] E. L. Ulrich, H. Akutsu, J. F. Doreleijers, Y. Harano, Y. E. Ioannidis, J. Lin, M. Livny, S. Mading, D. Maziuk, Z. Miller, E. Nakatani, C. F. Schulte, D. E. Tolmie, R. K. Wenger, H. Yao, J. L. Markley, *Nucleic Acids Res.* **2008**, *36*, D402–8.

[36] R. B. Best, J. Clarke, M. Karplus, *J. Am. Chem. Soc*. **2004**, *126*, 7734–5.

[37] (a) T. A. Wilkinson, M. V. Botuyan, B. E. Kaplan, J. J. Rossi, Y. Chen, *J. Mol. Biol.* **2000**, *303*, 515–29. (b) A. L. Olson, S. Cai, T. J. Herdendorf, H. M. Miziorko, D. S. Sem, *J. Am. Chem. Soc.* **2010**, *132*, 2102–3.

[38] F. A. A. Mulder, A. Mittermaier, B Hon, F. W. Dahlquist, L. E. Kay, *Nat. Struct. Biol.* **2001**, *8*, 932–5.

[39] B. A. Lyons, G. T. Montelione, *J. Magn. Reson., Ser. B* **1993**, *101*, 206–209.

[40] L. E. Kay, M. Ikura, R. Tschudin, A. Bax, *J. Magn. Reson.* **1990**, *89*, 496–514.

[41] Y. C. Li, G. T. Montelione, *J. Magn. Reson., Ser. B* **1994**, *105*, 45–51.

[42] K. Pervushin, R. Riek, G. Wider, K. Wüthrich, *Proc. Natl. Acad. Sci. U.S.A.* **1997**, *94*, 12366–71.
